# *N*′-[(*E*)-1-(5-Bromo-2-hy­droxy­phen­yl)ethyl­idene]-4-nitro­benzohydrazide

**DOI:** 10.1107/S1600536811023609

**Published:** 2011-06-25

**Authors:** Chang-Zheng Zheng, Liang Wang, Juan Liu, Yu-Jie Wang

**Affiliations:** a College of Environment and Chemical Engineering, Xi’an Polytechnic University, 710048 Xi’an,Shaanxi, People’s Republic of China

## Abstract

The title compound, C_15_H_12_BrN_3_O_4_, displays a *trans* conformation with respect to the C=N double bond. The central atoms around the C=N double bond are not coplanar, in contrast to the aromatic rings, which exhibit a dihedral angle of 1.80 (4)° between their mean planes. An intra­molecular O—H⋯N hydrogen bond occurs. In the crystal, mol­ecules are connected *via* inter­molecular N—H⋯O hydrogen bonding into chains along the *a* axis.

## Related literature

For the coordination properties of aroylhydrazones, see: Ali *et al.* (2004 ▶); Carcelli *et al.* (1995 ▶); Zhang *et al.* (2011 ▶); Zheng *et al.* (2008 ▶). 
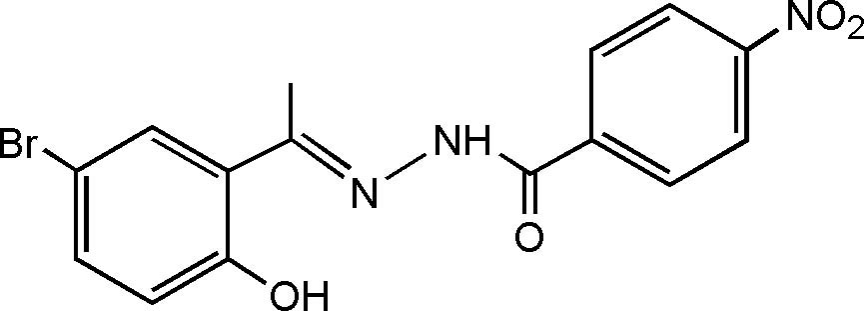


## Experimental

### 

#### Crystal data

C_15_H_12_BrN_3_O_4_
*M* *_r_* = 378.19
Orthorhombic, 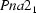

*a* = 40.381 (13) Å
*b* = 5.0598 (16) Å
*c* = 7.168 (2) Å
*V* = 1464.5 (8) Å^3^
*Z* = 4
Mo *K*α radiation
μ = 2.83 mm^−1^
*T* = 298 K
0.35 × 0.23 × 0.14 mm

#### Data collection

Bruker SMART CCD area-detector diffractometer
Absorption correction: multi-scan (*SADABS*; Sheldrick, 1996 ▶) *T*_min_ = 0.441, *T*_max_ = 0.689
6753 measured reflections
2520 independent reflections
2074 reflections with *I* > 2σ(*I*)
*R*_int_ = 0.040

#### Refinement

*R*[*F* ^2^ > 2σ(*F* ^2^)] = 0.049
*wR*(*F* ^2^) = 0.157
*S* = 0.95
2520 reflections
210 parameters
1 restraint
H-atom parameters constrained
Δρ_max_ = 0.95 e Å^−3^
Δρ_min_ = −0.75 e Å^−3^
Absolute structure: Flack (1983), 1075 Friedel pairs
Flack parameter: 0.01 (2)

### 

Data collection: *SMART* (Bruker, 1996 ▶); cell refinement: *SAINT* (Bruker, 1996 ▶); data reduction: *SAINT*; program(s) used to solve structure: *SHELXS97* (Sheldrick, 2008 ▶); program(s) used to refine structure: *SHELXL97* (Sheldrick, 2008 ▶); molecular graphics: *SHELXTL* (Sheldrick, 2008 ▶); software used to prepare material for publication: *SHELXTL*.

## Supplementary Material

Crystal structure: contains datablock(s) I, global. DOI: 10.1107/S1600536811023609/zq2106sup1.cif

Structure factors: contains datablock(s) I. DOI: 10.1107/S1600536811023609/zq2106Isup2.hkl

Supplementary material file. DOI: 10.1107/S1600536811023609/zq2106Isup3.cml

Additional supplementary materials:  crystallographic information; 3D view; checkCIF report

## Figures and Tables

**Table 1:** Hydrogen-bond geometry (Å, °)

*D*—H⋯*A*	*D*—H	H⋯*A*	*D*⋯*A*	*D*—H⋯*A*
N2—H2*A*⋯O2^i^	0.86	2.23	2.981 (6)	146
O1—H1⋯N1	0.82	1.81	2.531 (7)	145

Symmetry code: (i) 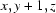.

**Fig. 1.**
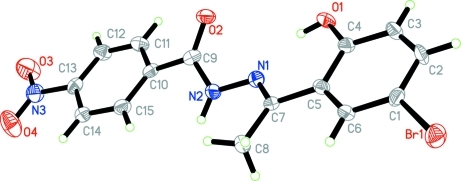
The molecular structure of the title compound showing the atom-numbering scheme and displacement ellipsoids drawn at the 30% probability level. H atoms are presented as small spheres of arbitrary radius.

**Fig. 2.**
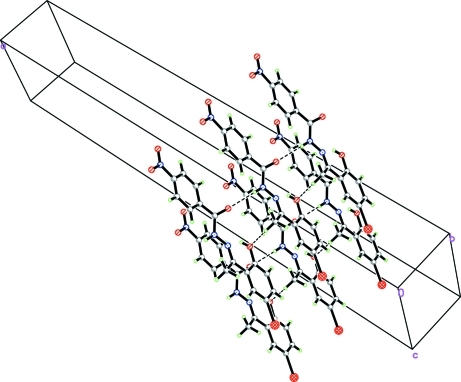
The crystal packing of the title compound viewed along the a axis. Dashed lines show intra- and intermolecular hydrogen bonds.

## supplementary crystallographic information

### Comment

The chemistry of aroylhydrazones continues to attract much attention due to their coordination ability to metal ions (Zhang *et al.*, 2011; Zheng *et al.*, 2008; Ali *et al.*, 2004) and their biological activity (Carcelli *et al.*, 1995). As an extension of work on the structural characterization of aroylhydrazone derivatives, the title compound, C_15_H_12_N_3_O_4_Br, was synthesized and its crystal structure is reported here.

The title compound, C_15_H_12_N_3_O_4_Br, displays a *trans* conformation with respect to the C=N double bond (Fig. 1). The central atoms around the C=N double bond are not coplanar since the dihedral angle C7—N1—N2—C9 is 154.7 (5)° in contrast to the aromatic rings which exhibit a dihedral angle of 1.80 (4)° between their mean planes. In the crystal structure, one intramolecular O—H···N hydrogen bond occurs (Table 1). The molecules are connected via intermolecular N—H···O into one-dimensional linear chains along the *a* axis (Table 1; Fig. 2).

### Experimental

Ethyl 4-nitrobenzoate (9.76 g, 0.05 mol) was dissolved in ethanol (40 ml) at room temperature and heated at 363 K, followed by the addition of hydrazine hydrate (2.50 g, 0.05 mol). Subsequently, the mixture was refluxed for 10 h, and then cooled to room temperature. The crystals were precipitated and collected by filtration. The product was recrystallized from ethanol and dried under reduced pressure to give compound of 4-nitrobenzhydrazide. 4-Nitrobenzhydrazide (4.53 g, 0.025 mol) was dissolved in ethanol (20 ml) at room temperature and heated at 363 K, followed by the addition of 5-bromo-2-hydroxyphenyl ethyl ketone (5.38 g, 0.025 mol). Subsequently, the mixture was refluxed for 9 h, and then cooled to room temperature. The crystals were precipitated and collected by filtration. The product was recrystallized from ethanol and dried under reduced pressure to the title compound.

### Refinement

All H atoms were positioned geometrically and treated as riding on their parent atoms, with *C*—*H* (methyl) = 0.96 Å, C—H (aromatic) = 0.93 Å, O—H = 0.82 Å, N—H = 0.86 Å and with *U*_iso_(H) = 1.5*U*_eq_(C_methyl_, O) and 1.2*U*_eq_(C_aromatic_, N).

### Crystal data

**Table:**

C_15_H_12_BrN_3_O_4_	*F*(000) = 760
*M**_r_* = 378.19	*D*_x_ = 1.715 Mg m^−^^3^
Orthorhombic, *P**n**a*2_1_	Mo *K*α radiation, λ = 0.71073 Å
Hall symbol: P 2c -2n	Cell parameters from 2077 reflections
*a* = 40.381 (13) Å	θ = 3.0–23.4°
*b* = 5.0598 (16) Å	µ = 2.83 mm^−^^1^
*c* = 7.168 (2) Å	*T* = 298 K
*V* = 1464.5 (8) Å^3^	Block, red
*Z* = 4	0.35 × 0.23 × 0.14 mm

### Data collection

**Table:**

Bruker SMART CCD area-detector diffractometer	2520 independent reflections
Radiation source: fine-focus sealed tube	2074 reflections with *I* > 2σ(*I*)
graphite	*R*_int_ = 0.040
φ and ω scans	θ_max_ = 25.2°, θ_min_ = 2.0°
Absorption correction: multi-scan (*SADABS*; Sheldrick, 1996)	*h* = −39→48
*T*_min_ = 0.441, *T*_max_ = 0.689	*k* = −6→6
6753 measured reflections	*l* = −7→8

### Refinement

**Table:**

Refinement on *F*^2^	Hydrogen site location: inferred from neighbouring sites
Least-squares matrix: full	H-atom parameters constrained
*R*[*F*^2^ > 2σ(*F*^2^)] = 0.049	*w* = 1/[σ^2^(*F*_o_^2^) + (0.120*P*)^2^ + 0.2524*P*] where *P* = (*F*_o_^2^ + 2*F*_c_^2^)/3
*wR*(*F*^2^) = 0.157	(Δ/σ)_max_ = 0.002
*S* = 0.95	Δρ_max_ = 0.95 e Å^−^^3^
2520 reflections	Δρ_min_ = −0.75 e Å^−^^3^
210 parameters	Extinction correction: *SHELXL97* (Sheldrick, 2008)
1 restraint	Extinction coefficient: 0.0113 (15)
Primary atom site location: structure-invariant direct methods	Absolute structure: Flack (1983), 1075 Friedel pairs
Secondary atom site location: difference Fourier map	Flack parameter: 0.01 (2)

### Special details

**Table:**

Geometry. All e.s.d.'s (except the e.s.d. in the dihedral angle between two l.s. planes) are estimated using the full covariance matrix. The cell e.s.d.'s are taken into account individually in the estimation of e.s.d.'s in distances, angles and torsion angles; correlations between e.s.d.'s in cell parameters are only used when they are defined by crystal symmetry. An approximate (isotropic) treatment of cell e.s.d.'s is used for estimating e.s.d.'s involving l.s. planes.
Refinement. Refinement of *F*^2^ against ALL reflections. The weighted *R*-factor *wR* and goodness of fit *S* are based on *F*^2^, conventional *R*-factors *R* are based on *F*, with *F* set to zero for negative *F*^2^. The threshold expression of *F*^2^ > σ(*F*^2^) is used only for calculating *R*-factors(gt) *etc*. and is not relevant to the choice of reflections for refinement. *R*-factors based on *F*^2^ are statistically about twice as large as those based on *F*, and *R*- factors based on ALL data will be even larger.

### Fractional atomic coordinates and isotropic or equivalent isotropic displacement parameters (Å^2^)

**Table:**

	*x*	*y*	*z*	*U*_iso_*/*U*_eq_	
Br1	0.748669 (14)	0.63795 (13)	0.8107 (3)	0.0527 (3)	
N1	0.63083 (12)	0.3604 (9)	0.2325 (8)	0.0407 (12)	
N2	0.61516 (12)	0.4290 (9)	0.0655 (7)	0.0398 (12)	
H2A	0.6165	0.5855	0.0194	0.048*	
N3	0.53035 (13)	0.4393 (12)	−0.7014 (9)	0.0537 (14)	
O1	0.63214 (10)	0.0383 (8)	0.5025 (7)	0.0454 (10)	
H1	0.6246	0.1078	0.4084	0.068*	
O2	0.59928 (11)	0.0029 (8)	0.0369 (7)	0.0534 (12)	
O3	0.50841 (18)	0.2902 (13)	−0.7491 (10)	0.094 (2)	
O4	0.53936 (16)	0.6248 (14)	−0.7978 (10)	0.087 (2)	
C1	0.71150 (13)	0.4542 (11)	0.7103 (8)	0.0383 (13)	
C2	0.69852 (14)	0.2403 (12)	0.8090 (10)	0.0460 (13)	
H2	0.7078	0.1873	0.9219	0.055*	
C3	0.67161 (18)	0.1098 (12)	0.7345 (10)	0.0477 (16)	
H3	0.6625	−0.0310	0.8000	0.057*	
C4	0.65783 (15)	0.1816 (11)	0.5655 (9)	0.0363 (13)	
C5	0.67081 (14)	0.4039 (10)	0.4651 (8)	0.0330 (12)	
C6	0.69784 (13)	0.5350 (10)	0.5410 (8)	0.0352 (12)	
H6	0.7070	0.6784	0.4781	0.042*	
C7	0.65665 (13)	0.4916 (10)	0.2846 (7)	0.0329 (12)	
C8	0.67260 (16)	0.7035 (12)	0.1733 (9)	0.0434 (15)	
H8A	0.6657	0.6895	0.0455	0.065*	
H8B	0.6962	0.6853	0.1804	0.065*	
H8C	0.6662	0.8728	0.2218	0.065*	
C9	0.59767 (14)	0.2338 (11)	−0.0194 (9)	0.0405 (14)	
C10	0.57928 (12)	0.3074 (10)	−0.1896 (10)	0.0353 (11)	
C11	0.55293 (16)	0.1454 (12)	−0.2431 (10)	0.0451 (17)	
H11	0.5465	0.0060	−0.1667	0.054*	
C12	0.53649 (15)	0.1907 (12)	−0.4076 (10)	0.0462 (15)	
H12	0.5186	0.0856	−0.4425	0.055*	
C13	0.54701 (15)	0.3955 (11)	−0.5200 (9)	0.0411 (14)	
C14	0.57239 (14)	0.5633 (11)	−0.4717 (9)	0.0410 (13)	
H14	0.5786	0.7022	−0.5492	0.049*	
C15	0.58848 (15)	0.5181 (11)	−0.3029 (9)	0.0432 (14)	
H15	0.6055	0.6298	−0.2655	0.052*	

### Atomic displacement parameters (Å^2^)

**Table:**

	*U*^11^	*U*^22^	*U*^33^	*U*^12^	*U*^13^	*U*^23^
Br1	0.0513 (4)	0.0578 (4)	0.0490 (4)	0.0046 (3)	−0.0137 (3)	−0.0030 (5)
N1	0.050 (3)	0.036 (3)	0.035 (3)	0.004 (2)	−0.007 (2)	0.000 (2)
N2	0.053 (3)	0.032 (2)	0.034 (3)	−0.004 (2)	−0.011 (2)	0.002 (2)
N3	0.055 (3)	0.057 (3)	0.050 (4)	0.001 (2)	−0.015 (3)	−0.001 (3)
O1	0.060 (3)	0.030 (2)	0.046 (3)	−0.0088 (19)	0.002 (2)	0.013 (2)
O2	0.077 (3)	0.030 (2)	0.054 (3)	−0.003 (2)	−0.018 (2)	0.001 (2)
O3	0.112 (4)	0.084 (4)	0.086 (6)	−0.029 (4)	−0.056 (4)	0.015 (3)
O4	0.079 (4)	0.115 (5)	0.067 (4)	−0.022 (3)	−0.021 (3)	0.032 (4)
C1	0.046 (3)	0.039 (3)	0.031 (3)	0.007 (2)	−0.004 (2)	−0.010 (3)
C2	0.066 (3)	0.047 (3)	0.025 (3)	0.011 (3)	−0.004 (3)	0.013 (3)
C3	0.072 (4)	0.038 (3)	0.033 (3)	0.005 (3)	0.006 (3)	0.012 (3)
C4	0.044 (3)	0.029 (3)	0.035 (3)	0.005 (2)	0.005 (2)	0.001 (2)
C5	0.037 (3)	0.028 (3)	0.034 (3)	0.005 (2)	0.006 (2)	0.004 (2)
C6	0.044 (3)	0.028 (3)	0.034 (3)	0.000 (2)	0.002 (2)	0.006 (2)
C7	0.044 (3)	0.023 (2)	0.031 (3)	0.003 (2)	0.001 (2)	−0.002 (2)
C8	0.056 (4)	0.041 (3)	0.033 (4)	−0.004 (3)	−0.005 (3)	0.005 (3)
C9	0.039 (3)	0.034 (3)	0.048 (4)	0.000 (3)	0.000 (3)	−0.009 (3)
C10	0.037 (2)	0.030 (2)	0.039 (3)	0.0031 (19)	−0.005 (3)	−0.009 (3)
C11	0.045 (3)	0.035 (3)	0.055 (5)	−0.004 (2)	0.001 (3)	0.004 (3)
C12	0.044 (3)	0.041 (3)	0.053 (4)	−0.007 (3)	−0.009 (3)	0.002 (3)
C13	0.040 (3)	0.044 (3)	0.040 (4)	0.003 (2)	−0.007 (2)	−0.012 (3)
C14	0.048 (3)	0.036 (3)	0.039 (4)	−0.002 (3)	−0.002 (3)	−0.002 (3)
C15	0.047 (3)	0.036 (3)	0.047 (4)	−0.004 (3)	−0.003 (3)	−0.005 (3)

### Geometric parameters (Å, °)

**Table:**

Br1—C1	1.906 (6)	C5—C6	1.388 (8)
N1—C7	1.291 (7)	C5—C7	1.483 (8)
N1—N2	1.397 (7)	C6—H6	0.9300
N2—C9	1.358 (7)	C7—C8	1.484 (8)
N2—H2A	0.8600	C8—H8A	0.9600
N3—O3	1.213 (8)	C8—H8B	0.9600
N3—O4	1.221 (8)	C8—H8C	0.9600
N3—C13	1.480 (8)	C9—C10	1.476 (9)
O1—C4	1.344 (8)	C10—C15	1.391 (9)
O1—H1	0.8200	C10—C11	1.396 (8)
O2—C9	1.238 (7)	C11—C12	1.373 (10)
C1—C6	1.394 (8)	C11—H11	0.9300
C1—C2	1.395 (9)	C12—C13	1.380 (9)
C2—C3	1.380 (10)	C12—H12	0.9300
C2—H2	0.9300	C13—C14	1.375 (8)
C3—C4	1.381 (10)	C14—C15	1.393 (9)
C3—H3	0.9300	C14—H14	0.9300
C4—C5	1.435 (8)	C15—H15	0.9300
			
C7—N1—N2	119.1 (5)	C5—C7—C8	121.2 (5)
C9—N2—N1	116.0 (5)	C7—C8—H8A	109.5
C9—N2—H2A	122.0	C7—C8—H8B	109.5
N1—N2—H2A	122.0	H8A—C8—H8B	109.5
O3—N3—O4	122.4 (7)	C7—C8—H8C	109.5
O3—N3—C13	119.1 (6)	H8A—C8—H8C	109.5
O4—N3—C13	118.5 (5)	H8B—C8—H8C	109.5
C4—O1—H1	109.5	O2—C9—N2	120.8 (6)
C6—C1—C2	121.3 (5)	O2—C9—C10	122.3 (5)
C6—C1—Br1	119.8 (4)	N2—C9—C10	116.7 (5)
C2—C1—Br1	118.9 (5)	C15—C10—C11	119.6 (6)
C3—C2—C1	118.1 (6)	C15—C10—C9	122.8 (5)
C3—C2—H2	121.0	C11—C10—C9	117.5 (5)
C1—C2—H2	121.0	C12—C11—C10	120.4 (6)
C2—C3—C4	122.1 (6)	C12—C11—H11	119.8
C2—C3—H3	119.0	C10—C11—H11	119.8
C4—C3—H3	119.0	C11—C12—C13	118.6 (6)
O1—C4—C3	117.6 (6)	C11—C12—H12	120.7
O1—C4—C5	122.4 (5)	C13—C12—H12	120.7
C3—C4—C5	119.9 (6)	C14—C13—C12	123.1 (6)
C6—C5—C4	117.7 (5)	C14—C13—N3	117.8 (6)
C6—C5—C7	120.1 (5)	C12—C13—N3	119.0 (5)
C4—C5—C7	122.1 (5)	C13—C14—C15	117.7 (6)
C5—C6—C1	120.8 (5)	C13—C14—H14	121.1
C5—C6—H6	119.6	C15—C14—H14	121.1
C1—C6—H6	119.6	C10—C15—C14	120.6 (5)
N1—C7—C5	114.2 (5)	C10—C15—H15	119.7
N1—C7—C8	124.5 (5)	C14—C15—H15	119.7
			
C7—N1—N2—C9	154.7 (5)	N1—N2—C9—O2	−8.8 (8)
C6—C1—C2—C3	−0.1 (9)	N1—N2—C9—C10	176.3 (5)
Br1—C1—C2—C3	179.9 (5)	O2—C9—C10—C15	−149.2 (6)
C1—C2—C3—C4	1.2 (10)	N2—C9—C10—C15	25.6 (8)
C2—C3—C4—O1	178.5 (6)	O2—C9—C10—C11	26.5 (8)
C2—C3—C4—C5	−2.2 (9)	N2—C9—C10—C11	−158.7 (5)
O1—C4—C5—C6	−178.7 (5)	C15—C10—C11—C12	1.0 (9)
C3—C4—C5—C6	1.9 (8)	C9—C10—C11—C12	−174.9 (5)
O1—C4—C5—C7	0.2 (8)	C10—C11—C12—C13	1.3 (10)
C3—C4—C5—C7	−179.2 (5)	C11—C12—C13—C14	−2.6 (10)
C4—C5—C6—C1	−0.8 (8)	C11—C12—C13—N3	177.5 (6)
C7—C5—C6—C1	−179.8 (5)	O3—N3—C13—C14	178.3 (7)
C2—C1—C6—C5	−0.1 (8)	O4—N3—C13—C14	−1.5 (9)
Br1—C1—C6—C5	179.9 (4)	O3—N3—C13—C12	−1.8 (10)
N2—N1—C7—C5	179.1 (4)	O4—N3—C13—C12	178.4 (7)
N2—N1—C7—C8	−4.0 (8)	C12—C13—C14—C15	1.5 (9)
C6—C5—C7—N1	−177.5 (5)	N3—C13—C14—C15	−178.6 (5)
C4—C5—C7—N1	3.6 (7)	C11—C10—C15—C14	−2.2 (9)
C6—C5—C7—C8	5.5 (8)	C9—C10—C15—C14	173.5 (5)
C4—C5—C7—C8	−173.4 (6)	C13—C14—C15—C10	0.9 (9)

### Hydrogen-bond geometry (Å, °)

**Table:**

*D*—H···*A*	*D*—H	H···*A*	*D*···*A*	*D*—H···*A*
N2—H2A···O2^i^	0.86	2.23	2.981 (6)	146.
O1—H1···N1	0.82	1.81	2.531 (7)	145.

Symmetry codes: (i) *x*, *y*+1, *z*.
